# The Muscle-Bone Unit in Male Elite Soccer Players Aged 14–19

**DOI:** 10.3390/jfmk10040432

**Published:** 2025-11-05

**Authors:** Valentina Cavedon, Carlo Zancanaro, Chiara Milanese

**Affiliations:** Laboratory of Anthropometry and Body Composition, Department of Neurosciences, Biomedicine and Movement Sciences, University of Verona, 37134 Verona, Italy; valentina.cavedon@univr.it (V.C.); chiara.milanese@univr.it (C.M.)

**Keywords:** dual-energy X-ray absorptiometry, sport, association football, adolescence

## Abstract

**Background**: Muscle and bone show reciprocal interactions and are associated in a muscle-bone unit. The muscle-bone unit has been investigated to a very limited extent in soccer players. The objective of this work was to investigate in detail the muscle-bone unit in male youth elite soccer players. **Methods**: Bone mineral and lean mass were measured with dual-energy X-ray absorptiometry (DXA). The functional muscle-bone unit (fMBU) and the muscle-to-bone ratio (MBR) were calculated from the DXA output in a sample of players aged 14–19 (n = 193) playing in the youth squads of an Italian Serie A team. **Results:** Statistically significant (*p* < 0.05) correlations were found between lean mass variables and bone mineral content and density, also after adjusting for age, body mass, stature, maturity, and ethnicity (White/Black). fMBU and MBR were statistically significantly associated with age, body mass, stature, maturity, and ethnicity. Linear regression showed that body lean mass was the strongest predictor for bone mineral content and density. Age was a statistically significant predictor for fMBU and MBR. Playing position did not show any statistically significant relationship with bone mineral content and density, as well as fMBU or MBR. Centiles for fMBU and MBR were calculated as a reference. **Conclusions**: This work is the first detailed characterization of the muscle-to-bone relationship in soccer players. It is expected to be of use for sport scientists and the wide community of sportsmen and professionals involved in soccer.

## 1. Introduction

Muscle and bone are closely associated since early development and represent a single functional system. They derive from the same progenitors [1], work in strict interdependence, forming the muscle-bone unit [2], and affect each other both anatomically and biochemically in a muscle-bone crosstalk [3]. Initially, the crosstalk between muscle and bone was attributed essentially to mechanotransduction. Further work showed that bone and muscle are secretory tissues, which are able to interact with one another using myokines and osteokines affecting distant bones and muscles, respectively. Physical activity plays a crucial role in muscle-bone crosstalk [4,5], essentially through mechanical stress [6], since the forces produced by skeletal muscles are more prominent than those associated with gravity [7], although exercise also has a potential to affect myokines and osteokines.

It has been shown that sports practice exerts a powerful osteogenic stimulus [8], especially during growth [9]. Soccer is a dynamic sport [10] characterized by high-intensity intermittent efforts involving high loading impact at body weight on the bone, e.g., rapid accelerations and decelerations, frequent changes in direction, and frequent jumps. In adults, soccer participation at a professional level has been shown to improve bone quality compared to nonsporting controls [11] and a different body weight-loading sport, such as elite running [12]. In youth, soccer has been shown to exert a positive effect on both bone mineral content (BMC) and bone mineral density (BMD) [13] even after controlling for several confounding variables [14]. Further, soccer may be superior to other sports in accruing bone mineral [13,15]. Besides being a key period for bone formation [16], adolescence, i.e., the period of maximal growth velocity, is also crucial to muscle mass accrual [17]. There is evidence that body lean soft tissue, mainly skeletal muscle mass, is a mediator of improved bone mineral density in sporting adolescents [18]. However, the status of the muscle-bone unit in youth elite soccer players has been investigated to a very limited extent [19].

When investigating the muscle-bone unit in a sporting population of growing individuals, maturity should be considered, as people of the same chronological age showing differences in stature and body mass [20] are comprised therein. In the context of youth soccer, the accurate determination of maturity through sophisticated laboratory techniques is not practical. The maturity offset (MO) [21] is a practical tool to reliably estimate maturity from simple anthropometric measurements [MO = −7.999994 + (0.0036124 x (age x height))] [22] and has been used in different sporting populations [23,24]; the equation estimates time before or after peak height velocity. Ethnicity (Eth) should also be considered when investigating the muscle–bone relationship in soccer players because of its capacity to affect body composition, including BMC and aBMD [25,26,27,28], and the muscle-bone unit [29]. Furthermore, the playing position (PP) may induce differences in the physiological and metabolic demands placed on the player that are distinctive to the position [30], thereby affecting body composition and, possibly, the muscle–bone relationship.

Dual-energy X-ray absorptiometry (DXA) is a reliable technique to assess BMC and BMD in youth [31], yielding, at the same time, accurate measurement of fat-free soft tissue mass (FFSTM). In general terms, the DXA outcome measures of bone mineral and lean (muscle) mass have been used as surrogates of bone and muscle strength, respectively [2].

Several DXA-derived body lean mass variables and indices could be used as determinants of bone mineral status. The Appendicular (upper limbs + lower limbs) FFSTM has been shown to represent a reliable proxy for body skeletal muscle mass [32,33], which, in turn, is associated with bone mineral [34,35,36] in the muscle-bone unit. The total body less head (TBLH) FFSTM incorporates Appendicular FFSTM and trunk lean soft tissue, representing a proxy of body mass, which, in turn, is associated with bone mineral status [37,38]. The stature-normalized FFSTM (fat-free mass index, FFMI = FFSTM (kg)/stature^2^ (m)) represents an absolute value, which is useful to compare individuals and groups [39,40] and is associated with bone mineral status, especially during growth [41].

Several DXA-derived variables have been used to assess the muscle–bone relationship. The functional muscle-bone unit (fMBU, BMC [g]/FFSTM [g]) [42] “could reflect the mass-related aspect of the mechanical equilibrium achieved by the system [i.e., the bone-muscle unit] as a function of its strain-related point” [43]. A similar index, the muscle-to-bone ratio (MBR, FFSTM/BMC), has been used to evaluate the relationships between lean muscle mass and bone mass in athletes [44,45,46]. Total body MBR has been previously explored in soccer players [47,48] using anthropometry and equations. In this work, we chose to use DXA for the estimation of MBR because it allows for a more direct measurement of muscle- and bone-related variables vs. anthropometry, also at the regional level.

In this cross-sectional study, a sample of youth elite soccer players was recruited to define the relationship between muscle mass and bone characteristics at the organismic and regional levels and to explore the effect thereupon of relevant variables such as chronological age, maturity, Eth, and PP.

## 2. Materials and Methods

### 2.1. Participants

A convenience sample of male elite soccer players aged 14–19 years was collected over several years from the youth squads of a club competing in the Italian Serie A for multiple investigations. Players trained 4/5 times a week for two hours (<18 y) or 5/6 times a week for two hours (<20 y) and had a championship match one time a week during the season. The study was conducted according to the Helsinki Declaration and approved by the local ethics committee (2019-UNIVRCLR-0422326). The sample size for linear regression was calculated a priori with G*Power 3.1 [49]. Setting a medium effect size (Cohen’s f2) of 0.15 with alpha = 0.01, power = 0.95, and a maximum number of predictors = 5, the calculated total sample size was n = 180. Where needed, participants were subdivided into the following PPs: Goalkeeper (GK, n = 19), Defender (D, n = 60), Midfielder (M, n = 76), and Forward (F, n = 38). Considering the aims of this work, further fractionation of the study sample into sub-positions, e.g., central and lateral defender, was deemed not essential. All data were collected in the summer, before starting conditioning for the competitive season. The inclusion criteria were as follows: 14 y < age <20 y, no acute illness of any kind, no major injuries in the last six months, and no medications potentially affecting body composition or bone in the previous twelve months.

### 2.2. Procedures

Body mass was measured using an electronic scale (Tanita electronic scale BWB-800 MA, Wunder SA.BI. Srl, Milano, Italy) and recorded to the nearest 0.1 kg. A Harpenden stadiometer (Holtain Ltd., Crymych, Pembs, UK) was used to measure stature at the nearest mm of the participant without shoes and with minimal clothing. The body mass index (BMI) was calculated as weight (kg)/height (m^2^). For body composition evaluation, FFSTM, BMC, and areal BMD (aBMD) were measured using a total body DXA scanner (QDR Horizon, Hologic, Marlborough, MA, USA; fan-beam technology, software version 13.6.05). Possible baseline drift was assessed daily against a reference phantom supplied by the manufacturer. Scans were performed in the late morning in a post-absorptive state. Participants were instructed to avoid vigorous exercise for at least 24 h before measurement. The protocol of Nana et al. [50] was adopted for measurements. All other procedures were according to the manufacturer’s recommendations. All scans were taken of the whole body (WB). In vivo short-term precision was calculated by repeated (n = 2) scanning of 30 subjects with repositioning [51]; precision (percent coefficient of variation) was 0.59%, 0.75%, and 0.68% for FFSTM, BMC, and aBMD, respectively. The Hologic software provides readings for the WB and the trunk, the entire arm (left and right), the entire leg (left and right), and the head. In this work, BMC and aBMD of the total body less head region (TBLH) was considered in the analysis because the skull contains a large amount of total body mineral and is insensitive to physical activity [52]. The WB values for BMC and aBMD were also reported to compare with the previous literature. FFSTM, BMC, and aBMD were also calculated for the upper limbs together (Arms), lower limbs together (Legs), and the four limbs together (Appendicular). The same operator performed all scans and regional measurements to ensure consistency. The fMBU was expressed as fMBU_tot_, i.e., the ratio of TBLH BMC to WB FFSTM [53,54], and fMBU_app_, i.e., the ratio of Appendicular BMC to Appendicular FFSTM. The MBR was calculated at the WB and regional (Arms, Legs, and Trunk) level.

Stature and body mass may represent confounding variables for bone measurements because DXA is a projectional technique. DXA-derived measurements do not adequately correct for body and/or bone size [55]. Accordingly, the bone mineral was also expressed as bone mineral apparent density (BMAD, g/cm^3^) according to the formula proposed by Katzman et al. [56]: BMAD = BMC/(bone area^2^/body stature), thereby reducing the risk of overestimating players with higher stature and the risk of underestimating those with shorter stature [56].

Fractional age (FA) was calculated (YEARFRAC function in Microsoft Excel) and used throughout the analysis to fully exploit the information potential of chronological age in a sample of growing individuals.

### 2.3. Statistical Analysis

The normality of data was assessed using the Shapiro–Wilk test. Data are presented as mean ± standard deviation. Correlation and partial correlation (PC) analysis was conducted by calculating Pearson’s r and r_(PC)_. The strength of correlation was rated as per Hopkins [57], i.e., small (0–0.30), moderate (0.31–0.49), large (0.50–0.69), very large (0.70–0.89), and almost perfect (0.90–1).

One-way ANOVA in the general linear model was used to assess differences in fMBU and MBR in the four PPs (GK, D, M, F) and the effect of covariates. The Levene test assessed homogeneity of variance. Heteroscedasticity was evaluated with the Breusch–Pagan test. In case of significance, post hoc analysis with Bonferroni’s correction was conducted to assess differences between individual PPs. The effect size (partial η^2^) was calculated and rated according to Cohen [58] as small (0.01), medium (0.06), and large (0.14).

Linear regression analysis was carried out with bone mineral variable as the dependent variable and TBLH FFSTM, Appendicular FFSTM, or FFMI as the independent variable, together with FA, MO, Eth, and PP. The goodness-of-fit of models were assessed by calculating the adjusted coefficient of determination (AdjR^2^) and the standard error of the estimate (SEE). The standardized partial regression coefficient β assessed the independent effect of each predictive variable in bivariate regression. All covariates and predictors were selected after checking for multicollinearity.

Statistical significance was set at *p* ≤ 0.05. The IBM SPSS statistical package (v. 26, Armonk, NY, USA) was used for all analyses.

## 3. Results

### 3.1. Characteristics of the Study Sample

The final dataset included 193 players; therefore, the study was enough powered. The mean age of participants was 17.3 ± 1.06 y. The mean body mass and stature were 71.6 ± 6.96 kg and 179.7 ± 6.14 cm, respectively. The mean BMI was 22.1 ± 1.57 kg/m^2^. Most participants were White (n = 172); the rest were Black (n = 21).

### 3.2. Correlation of Muscle and Bone Variables

**Figure 1:**
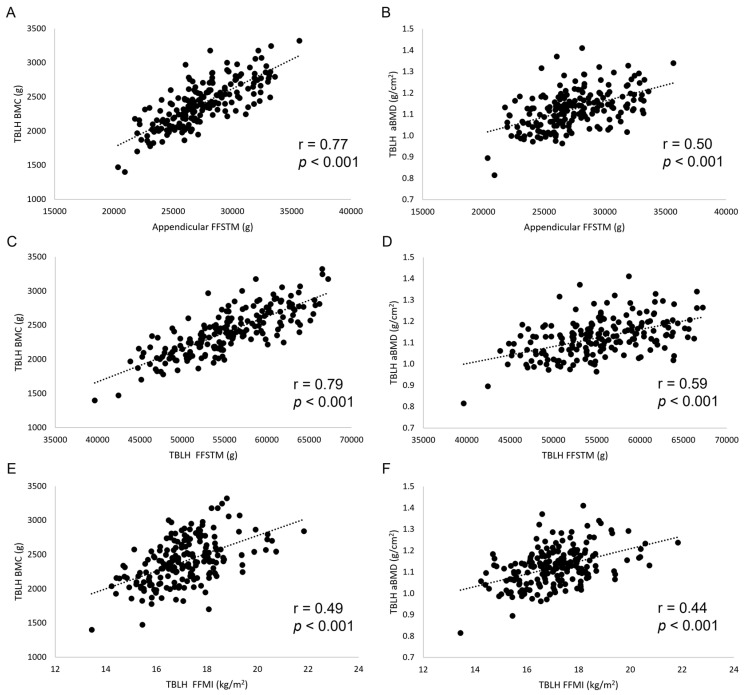
Scatterplots (**A**–**F**) showing the relationship between lean mass and bone mineral variables. TBLH, total body less head; BMC, bone mineral content.

The results of bivariate and adjusted correlation analysis between lean mass and bone variables are shown in Table 1 and Figure 1. Very large (r from 0.70 to 0.79), statistically significant correlations were found for Appendicular FFSTM and TBLH FFSTM, and WB, TBLH, Appendicular, and Arms BMC. Appendicular FFSTM and TBLH FFSTM showed large (r from 0.53 to 0.69), statistically significant correlations with Pelvis and Legs BMC and Arms aBMD. The correlation of FFMI with bone variables was moderate to large (r from 0.21 to 0.54), being statistically significant in most instances. No statistically significant correlation was found between Appendicular FFSTM, TBLH FFSTM, and FFMI (collectively “lean mass variables” from here on) and Appendicular and TBLH BMAD. Adjusting correlation for several confounding variables (body mass, stature, FA, Eth, and MO for lean mass variables and body mass, FA, Eth, and MO for BMAD) resulted in a general reduction in the r value. However, all the lean mass variables statistically significantly correlated with TBLH BMC, Appendicular BMC, and aBMD, and Arms and Legs BMC and aBMD. No statistically significant bivariate or adjusted correlation was found for BMAD.

The mean values for fMBU in the youth elite soccer players sample were as follows: fMBU_tot_ (g/g), 0.0410 ± 0.00351; fMBU_app_ (g/g), 0.0579 ± 0.00621. The mean values for MBR were as follows: MBR_WB_ (kg/kg), 23.3 ± 1.73; MBR_arms_ (kg/kg), 18.7 ± 1.59; MBR_legs_ (kg/kg), 17.1 ± 1.97; MBR_trunk_ (kg/kg), 35.1 ± 5.56.

Table 2 shows the results of correlation analysis between fMBU and MBR, and body mass, stature, MO, and Eth, as well as FA (for the latter, both bivariate and adjusted correlation coefficients are presented; see table for details).

**Figure 2:**
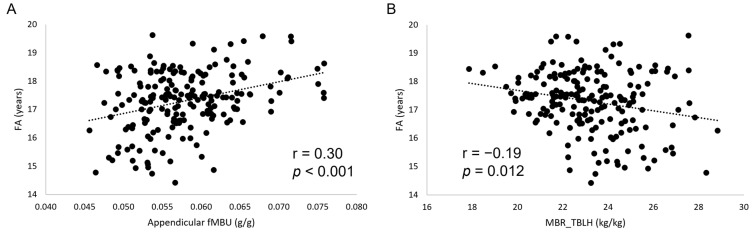
Scatterplots (**A**,**B**) showing the relationship between fractional age (FA) and muscle-bone indices. fMBU, functional muscle-bone index; MBR, muscle-to-bone ratio.

FA showed statistically significant, small to moderate correlations with all muscle-bone indices (Figure 2). A similar pattern was found for MO (except for MBR_trunk_) at a correlation strength from small to moderate. A decreasing number of statistically significant, small correlations was found for body mass, stature, and Eth. After adjusting for all the other biological variables, Eth was the only variable to show a small, statistically significant correlation with a muscle-bone unit index (fMBU_tot_: r = 0.168, *p* = 0.021; MBR_WB_: r = −0.172, *p* = 0.018).

### 3.3. Results of ANOVA

One-way ANOVA conducted for fMBU and MBR with PP as the main factor showed no statistically significant difference for any index (F_(3,192)_ from 0.192 to 1.089, *p* from 0.902 to 0.351, effect size from 0.003 to 0.017, observed power from 0.094 to 0.265). Similar results were obtained when the same analysis was carried out with FA, Eth, MO, body mass, and stature as covariates (Table 3). The effect size was medium for all variables.

### 3.4. Centiles for Bone-Muscle Indices

Centiles for all muscle-bone indices are presented in Table 4. Please note that data are presented of the White players only, because the number of Black players (n = 21) prevented meaningful calculation of centiles.

### 3.5. Results of Linear Regression Analysis

Results of linear regression analysis carried out in the whole sample (n = 193) using TBLH FFSTM, Appendicular FFSTM, and FFMI together with FA, MO, Eth, and PP as the predictors of bone mineral variables are summarized in Table 5, Table 6 and Table 7. All models were statistically significant (*p* ≤ 0.05). The Durbin–Watson statistic was <2.5, and the variance inflation factor was <5.0.

In regression analysis, TBLH FFSTM (Table 5) showed the highest, statistically significant β coefficient for a large majority of bone mineral variables in comparison with FA, MO, Eth, and PP; FA showed the highest, statistically significant β coefficient for some aBMD variables and Trunk BMC. Similar findings were found when Appendicular FFSTM was used in analysis (Table 6). When the stature-adjusted lean mass index FFMI was used in regression analysis (Table 7), MO showed the highest, statistically significant β coefficient for most bone mineral variables. FFMI and FA showed the highest, statistically significant β coefficient for a few aBMD and BMAD variables, respectively. PP showed not statistically significant β coefficients.

Regression analysis carried out for functional muscle-bone indices with FA, body mass, stature, Eth, and PP as the independent variables yielded the results presented in Table 8. All models were statistically significant (*p* ≤ 0.05). The Durbin-Watson statistic was always <2.5, and the variance inflation factor was <5.0.

FA was the only predictor showing a statistically significant β coefficient for all indices. Eth showed a statistically significant β coefficient for fMBU_WB_ and MBR_TBLH_, both of which were lower than those for FA. Stature, body mass, and PP showed no statistically significant β coefficient for all indices.

## 4. Discussion

In this work, we investigated the muscle-bone unit in a large sample of youth elite soccer players by means of DXA to characterize the functional relationship of muscle and bone mineral mass and density in this sporting population and the possible effect on it of several confounding biological variables.

The first finding of this work was that, in youth elite soccer players, BMC and aBMD statistically significantly correlate with body lean mass at the WB, TBLH, and regional levels. While a positive relationship between lean mass and bone is well documented [59] and may be easily explained by their consensual accretion during growth, information on the correlation between lean mass and bone mineral mass or density is scant. Capozza et al. [60] found a correlation (r = 0.71) between lean mass and BMC at WB in males, which is superimposable to that found in the current study (Table 1). In another study [61] it was found that, in young adults, a statistically significant correlation exists between FFMI and WB BMC (r = 0.61), aBMD (r = 0.60), LS BMC (r = 0.35), and LS BMD (r = 0.33), which is consistent with findings in this work (Table 1). As expected, the correlation of bone variables with the stature-adjusted index FFMI was generally lower than that shown by absolute lean mass variables (TBLH FFSTM and Appendicular FFSTM) because stature is in a linear relationship with muscle mass [62]. After adjusting for several confounding factors (Table 1), the correlation between TBLH BMC, Appendicular BMC, and aBMD, and Appendicular FFSTM, TBLH FFSTM, and FFMI remained statistically significant, suggesting an independent reciprocal influence between bone and lean mass in the studied population. The lack of statistically significant bivariate and adjusted correlation between BMAD and lean mass (Table 1) further underlines the role of stature in mediating the relationship between lean mass and bone mineral.

The results of the correlation analysis presented in Table 2 showed a small, statistically significant r value between biological variables (body mass, stature, MO, Eth, and FA) and fMBU (mind that fMBU_tot_ is calculated as TBLH BMC/WB FFSTM to avoid the confounding effect of head BMC, which represents a large proportion of WB BMC and may increase in a non-linear relationship with TBLH BMC), as well as MBR. These findings indicate that, at the organismic level, chronological age, body size, and maturity are associated with an increase in the amount of BMC accrued per unit lean mass. Interestingly, Eth was also positively associated with both muscle-bone indices. Since players participating in this study were White and Black (coded as “dummy” variables in the dataset), it is apparent that Black ethnicity plays a role in increasing the amount of BMC accrued per unit lean mass in youth elite soccer players. This point will be further discussed in the following. At the regional (Appendicular, Trunk, Arms, And Legs) level, FA showed a statistically significant r value for all muscle-bone indices (4/4), followed by MO (3/4), and body mass and stature (2/4). These data suggest that among the considered biological variables, chronological age is the only one able to act on the amount of BMC accrued per unit lean mass at all sites in the body. Playing position, a soccer-specific variable, showed no statistically significant correlation with any muscle-bone index. Therefore, PP seems not involved in mediating BMC accrual per unit lean mass in this sporting population. This conclusion is supported by ANOVA for muscle-bone indices conducted with PP as the factor and FA, body mass, stature, MO, and Eth as covariates (Table 3), showing no statistically significant difference for any index. Regression analysis (Table 8) confirmed that FA is the main factor determining the muscle-bone index at the organismic and regional level, with a minor role played by Eth at the organismic level only, whereas no significant role is played by body size (body mass and stature) or PP.

Table 4 presents the centiles for the muscle-bone indices in the White players. The age distribution of participants prevented the calculation of meaningful centiles for year age. However, it should be considered that the sample of White players was relatively homogeneous in terms of body size (mean BMI = 22.1 ± 1.55 kg/m^2^), and all participants showed positive MO values (mean: 3.2 ± 0.79 y). Given the lack of an established optimum range of muscle-bone indices in soccer players, the presented data can be practical for both athletes and coaches.

The relative importance of each lean mass variable (TBLH FFSTM, Appendicular FFSTM, and FFMI) and other variables of interest (FA, MO, Eth, and PP) in predicting bone mineral variables was assessed in linear regression analysis by calculating the standardized partial regression coefficient β. Considering the statistically significant β coefficients in Table 5, Table 6 and Table 7, TBLH FFSTM showed the highest values in comparison with Appendicular FFSTM and FFMI. Appendicular FFSTM, in turn, showed higher values than FFMI. This indicates that the overall lean mass (Appendicular + Trunk) and not skeletal muscle mass or stature-normalized WB lean mass better explain BMC and aBMD. This is possibly due to the positive action body mass exerts, per se, on bone mineral [37]. The role of lean mass in determining bone mineral variables in youth soccer players is confirmed by previous findings in Brazilian male players who were 12 to 18 y of age (n = 148) [63], where DXA-measured lean mass was the most important predictor for TBLH BMC (R^2^ = 0.524), LS BMC (R^2^ = 0.492), and LS BMD (R^2^ = 0.513). In the present study, FA showed some statistically significant β coefficients in the models, including TBLH FFSTM and Appendicular FFSTM. Most β coefficients for FA were higher than those for TBLH, FFSTM, and Appendicular FFSTM in predicting aBMD at several sites. This is consistent with the soccer players still growing [54]. Instead, in the model with FFMI, FA showed higher β coefficients values than FFMI for almost all BMC variables. Since FFMI is a stature-adjusted index, this reveals a relevant stature-independent role of chronological age in bone mineral accrual in growing elite soccer players, as confirmed by previous findings in the general population [28]. Maturity offset, which incorporates the product of age and stature, showed a limited number of statistically significant β coefficients in the models, including TBLH FFSTM, or Appendicular FFSTM. In both models, MO showed the highest β coefficient among all predictive variables for Trunk BMC and aBMD. In the model including FFMI, MO showed the highest β coefficient for most BMC and aBMD variables, while FA showed negative coefficients. This indicates that MO should be given attention when investigating the relationships between bone mineral variables and lean mass variables in growing soccer players. Eth showed several low, albeit statistically significant, positive β coefficients, especially in the model including TBLH FFSTM. This finding could be explained by the higher average lean mass found in Black vs. White people [64]. A previous work using DXA [65] did not find a statistically significant difference in lean mass between Caucasian and non-Caucasian adult elite soccer players. However, in the study of Sutton et al. [65], the non-Caucasian group included players of African-Caribbean and Asian descent and mixed race, which may be non-comparable with the Black players investigated in the present work. PP did not show any statistically significant β coefficient in any model, showing that the different positions on the field do not affect, per se, the muscle-bone unit. A difference in body composition is often found in soccer players between goalkeepers and outfield players, both in adult [65] and youth soccer players [47,65,66]. However, such a difference typically involves FM and %FM, thereby limiting its impact on bone mineral variables.

The mean value of fMBU_tot_ was in line with those found in the adolescent general population [43]. This may be due, at least in part, to the fact that the ratio between two variables (muscle mass and skeletal mass) growing roughly in a similar way tends to be similar across a large interval of values. However, the mean fMBU_app_ value was closer to that of young adults in the general population [43], suggesting that elite soccer practice is highly effective in promoting bone mineral accrual per unit muscle mass in the limbs of youth players. This finding is supported by previous findings [13] showing increased BMC and BMD in youth soccer players. Interestingly, the mean MBR values found in elite youth soccer players at TBLH, Arms, Legs, and Trunk were obviously higher than in 20-year-old track and field athletes [46], as well as younger (20.1 y) [67] and older (24.2 y) [44] American football players, suggesting that those sports promote lower and higher bone mineral accrual per unit muscle mass vs. soccer, respectively. Estimation of β coefficients in linear regression (Table 8) showed a main role for FA in determining fMBU and MBR. This finding is supported by data in the general healthy population showing a steady increase in fMBU in male adolescents [68]. Interestingly, Eth showed statistically significant β coefficients for fMBU_tot_ and MBR_WB_, indicating that Eth is able to differentially modulate the age-associated changes in the muscle-bone unit. Previous work in the general population [29] (age: 5–35 y) showed no differences in the muscle-bone unit according to race. However, participants in the current study were highly fitted sporting adolescents and it is reasonable to infer that an effect of Eth on the muscle-bone unit only emerges upon long periods of intense physical activity. Further investigation with a larger number of soccer players is needed to confirm such a hypothesis. Playing position did not show a statistically significant effect on any index (Table 8), confirming the results presented in Table 5, Table 6 and Table 7 and suggesting that soccer practice is associated with similar accumulation of bone mass per unit increase in muscle mass independently of the role played on the field. This suggestion is supported by findings obtained at the WB level using anthropometry by Bernal-Orozco et al. [47] showing very limited statistically significant differences among PPs in young (<20 y) Mexican soccer players.

This paper has strengths that should be underlined. First, to the best of our knowledge, this paper is the first to present both WB and regional data on the muscle-bone unit in soccer players. The study included a large number of participants, thereby offering a broad overview of muscle-bone characteristics with the production of reference data. Second, the use of DXA for body composition analysis guaranteed accurate measurement of the lean and bone mineral components at the WB and regional level, which is impossible using anthropometry. Third, the influence of PP on the muscle–bone relationship was explored.

This study has limitations. First, it was cross-sectional and not longitudinal, thereby limiting insights into the biology of the muscle-bone unit in adolescent elite soccer players. Second, dietary information was not available for the young participants that would have added sound information to the paper with special reference to mineral accrual. Third, performance metrics were not investigated, so the association of the muscle-bone unit and sport-specific variables could not be assessed.

## 5. Conclusions

In conclusion, the novelty of this work is the extensive characterization of the relationship between lean mass and bone in youth elite soccer players. It was shown that both muscle mass and lean mass are independent determinants of the bone mineral content and density, albeit stature plays an important role in mediating such a relationship. fMBU and MBR were especially modulated by chronological age, and Black ethnicity showed a positive effect on BMC accrual per unit lean mass. Reference values for fMBU and MBR were provided. The playing position did not affect the muscle-to-bone ratio to any extent. Findings presented herein should be of use for evaluating and monitoring youth soccer players.

## Figures and Tables

**Table 1:** Bivariate and partial correlations between Appendicular fat-free soft tissue mass (App FFSTM, g), total body less head (TBLH) FFSTM (g), and fat-free mass index (FFMI, g/cm^2^) and bone mineral variables in 193 male youth elite soccer players.

	Correlation Coefficient (Pearson’s r)					
Bone Mineral Variable	Lean Mass Variable					
	App FFSTM		TBLH FFSTM		FFMI	
	Bivariate	Adjusted ^	Bivariate	Adjusted ^	Bivariate	Adjusted ^
WB BMC (g)	0.70 **	0.14	0.71 **	0.17 *	0.48 **	0.22 *
WB aBMD (g/cm^2^)	0.38 **	0.03	0.36 **	0.04	0.39 **	0.08
TBLH BMC (g)	0.77 **	0.19 *	0.79 **	0.22 *	0.49 **	0.23 *
TBLH aBMD (g/cm^2^)	0.50 **	0.06	0.49 **	0.07	0.44 **	0.10
LS BMC (g)	0.45 **	0.13	0.48 **	0.21 *	0.31 *	0.21 *
LS aBMD (g/cm^2^)	0.36 **	0.09	0.36 **	0.12	0.35 **	0.13
Pelvis BMC (g)	0.65 **	0.14	0.69 **	0.23 **	0.47 **	0.23 **
Pelvis aBMD (g/cm^2^)	0.45 **	0.12	0.43 **	0.12	0.37 **	0.14
Trunk BMC (g)	0.47 **	−0.14	0.55 **	−0.02	0.21 *	−0.04
Trunk aBMD (g/cm^2^)	0.23 **	−0.13	0.26 **	−0.10	0.11	−0.11
Appendicular BMC (g)	0.74 **	0.31 **	0.72 **	0.26 **	0.41 **	0.29 **
Appendicular aBMD (g/cm^2^)	0.43 **	0.19 *	0.52 **	0.17 *	0.47 **	0.21 *
Arms BMC (g)	0.77 **	0.31 **	0.78 **	0.31 **	0.54 **	0.33 **
Arms aBMD (g/cm^2^)	0.53 **	0.49 **	0.54 **	0.31 **	0.48 **	0.33 **
Legs BMC (g)	0.69 **	0.27 **	0.67 **	0.22 *	0.49 **	0.25 **
Legs aBMD (g/cm^2^)	0.39 **	0.19 *	0.37 **	0.18 *	0.46 **	0.22 *
Appendicular BMAD (g/cm^3^)	−0.08	0.01	−0.09	0.04	0.12	0.07
TBLH BMAD (g/cm^3^)	−0.123	−0.11	−0.16	−0.14	−0.05	−0.11

*Legend:* WB, whole body; LS, lumbar spine; BMC, bone mineral content; aBMD, areal bone mineral density; BMAD, bone mineral apparent density; ^, DXA-measured variables are adjusted for body mass, stature, FA, Eth, and MO; stature-adjusted index (BMAD) is adjusted for body mass, FA, Eth, and MO; *, *p* < 0.05; **, *p* ≤ 0.001.

**Table 2:** Correlation coefficient between muscle-bone unit indices and body mass, stature, maturity offset (MO), ethnicity (Eth), and fractional age (FA). The adjusted correlation coefficient for FA is also shown.

Muscle-Bone Unit Index	Correlation Coefficient (Pearson’s r)					
	Body Mass	Stature	MO	Eth	FA	
					Bivariate	Adjusted ^
fMBU_tot_ (g/g)	0.28 **	0.24 **	0.32 **	0.20 *	0.24 **	0.11
fMBU_app_ (g/g)	0.14	0.10	0.33 **	0.10	0.34 **	0.11
MBR_WB_ (kg/kg)	−0.23 **	−0.21 *	−0.29 *	−0.20 *	−0.22 *	−0.13
MBR_arms_ (kg/kg)	−0.22 *	−0.21 *	−0.30 **	−0.13	−0.23 *	−0.13
MBR_legs_ (kg/kg)	−0.12	−0.09	−0.32 **	−0.10	−0.32 **	−0.14
MBR_trunk_ (kg/kg)	−0.15 *	−0.20 *	0.04	−0.13	0.16 *	0.01

*Legend:* ^, adjusted for body mass, stature, maturity offset, and ethnicity; *, *p* < 0.05; **, *p* ≤ 0.001.

**Table 3:** Results of one-way ANOVA conducted with PP as the main factor and fractional age, ethnicity, maturity offset, body mass, and stature as the covariates for functional muscle-bone unit (fMBU) at whole body (tot), total body less head (TBLH), and Appendicular (App), as well as muscle-to-bone ratio (MBR) at TBLH, upper limbs (Arms), lower limbs (Legs), and Trunk. Partial η^2^, effect size.

Variable	F Value	*p* Value	Partial η^2^
fMBU_tot_ (g/g)	0.693	0.558	0.011
fMBU_app_ (g/g)	0.614	0.607	0.010
MBR_WB_ (kg/kg)	0.590	0.622	0.010
MBR_arms_ (kg/kg)	0.600	0.616	0.010
MBR_legs_ (kg/kg)	0.785	0.504	0.013
MBR_trunk_ (kg/kg)	0.641	0.590	0.010

**Table 4:** Centiles for functional muscle-bone unit (fMBU) at whole body (tot), total body less head (TBLH), and Appendicular (App), as well as muscle-to-bone ratio (MBR) at TBLH, upper limbs (Arms), lower limbs (Legs), and Trunk in White youth elite soccer players.

Variable	Centile						
	5	10	25	50	75	90	95
fMBU_tot_ (g/g)	0.0350	0.0365	0.0384	0.009	0.0430	0.0454	0.0470
fMBU_app_ (g/g)	0.0489	0.0504	0.0533	0.0567	0.0609	0.0655	0.0711
MBR_WB_ (kg/kg)	20.23	20.86	22.000	23.24	24.59	25.01	26.89
MBR_arms_ (kg/kg)	16.10	16.75	17.57	18.83	19.80	21.09	21.91
MBR_legs_ (kg/kg)	13.28	14.40	16.08	17.31	18.61	19.81	20.35
MBR_trunk_ (kg/kg)	28.89	29.41	31.42	34.12	37.85	44.00	47.91

**Table 5:** Linear regression of total body less head (TBLH, g) and fat-free soft tissue mass (FFSTM, g) together with fractional age (FA), maturity offset (MO), ethnicity (Eth), and playing position (PP) on bone mineral variables in the whole sample of elite youth soccer players (n = 193). The standardized regression coefficient (β) and its *p* value, as well as the overall adjusted coefficient of determination (AdjR^2^) and standard error of the estimate (SEE), are reported. Non-statistically significant *p* values are in bold.

Variable	β Coefficient										AdjR^2^	SEE
	TBLH FFSTM	*p* Value	FA	*p* Value	MO	*p* Value	Eth	*p* Value	PP	*p* Value		
WB BMC (g)	0.581	<0.001	−0.024	**0.849**	0.223	**0.123**	0.152	0.003	0.038	**0.502**	0.553	241.9
WB aBMD (g/cm^2^)	0.349	<0.001	0.425	0.008	−0.226	**0.224**	0.238	<0.001	0.086	**0.232**	0.261	0.0799
TBLH BMC (g)	0.633	<0.001	−0.158	**0.146**	0.351	0.006	0.105	0.018	0.031	**0.528**	0.662	193.1
TBLH aBMD (g/cm^2^)	0.447	<0.001	0.301	0.049	−0.091	**0.606**	0.184	0.003	0.082	**0.230**	0.332	0.0729
Appendicular BMC (g)	0.606	<0.001	0.137	**0.257**	0.101	**0.472**	0.122	0.014	0.000	**0.995**	0.576	164.2
Appendicular aBMD (g/cm^2^)	0.416	<0.001	0.669	<0.001	−0.398	0.027	0.125	0.046	0.026	**0.703**	0.316	0.1032
Arms BMC (g)	0.675	<0.001	0.153	**0.175**	0.026	**0.843**	0.124	0.007	0.078	**0.123**	0.634	34.9
Arms aBMD (g/cm^2^)	0.503	<0.001	0.493	0.001	−0.250	**0.146**	0.087	**0.145**	−0.050	**0.456**	0.369	0.0471
Legs BMC (g)	0.554	<0.001	0.126	**0.340**	0.117	**0.444**	0.114	0.032	0.022	**0.705**	0.501	145.3
Legs aBMD (g/cm^2^)	0.398	<0.001	0.726	<0.001	−0.469	0.010	0.138	0.029	0.029	**0.678**	0.300	0.1466
LS BMC (g)	0.406	<0.001	−0.023	**0.887**	0.127	**0.504**	0.060	**0.363**	−0.012	**0.869**	0.225	12.8
LS aBMD (g/cm^2^)	0.333	<0.001	0.375	0.025	−0.181	**0.348**	0.170	0.012	0.053	**0.478**	0.204	0.1641
Pelvis BMC (g)	0.583	<0.001	−0.034	**0.798**	0.236	**0.123**	0.020	**0.703**	0.085	**0.151**	0.503	57.9
Pelvis aBMD (g/cm^2^)	0.394	<0.001	0.170	**0.285**	−0.025	**0.894**	0.220	0.001	0.116	**0.107**	0.267	0.1464
Trunk BMC (g)	0.389	<0.001	−0.590	<0.001	0.619	0.001	0.028	**0.650**	0.069	**0.313**	0.337	119.9
Trunk aBMD (g/cm^2^)	0.165	**0.102**	−0.281	**0.120**	0.348	**0.097**	0.050	**0.491**	0.100	**0.215**	0.064	0.1100
WB BMAD (g/cm^3^)	−0.305	0.002	0.100	**0.556**	0.123	**0.531**	0.269	<0.001	0.134	**0.080**	0.168	0.00544
TBLH BMAD (g/cm^3^)	−0.252	0.010	0.130	**0.445**	0.093	**0.644**	0.222	0.002	0.130	**0.098**	0.126	0.00590

WB, whole body; LS, lumbar spine; BMC, bone mineral content; aBMD, areal bone mineral density; BMAD, bone mineral apparent density.

**Table 6:** Linear regression of Appendicular fat-free soft tissue mass (App FFSTM, g) together with fractional age (FA), maturity offset (MO), ethnicity (Eth), and playing position (PP) on bone mineral variables in the whole sample of elite youth soccer players (n = 193). The standardized regression coefficient (β) and its *p* value, as well as the overall adjusted coefficient of determination (AdjR^2^) and standard error of the estimate (SEE), are reported. Non-statistically significant *p* values are in bold.

Variable	β Coefficient										AdjR^2^	SEE
	App FFSTM	*p* Value	FA	*p* Value	MO	*p* Value	Eth	*p* Value	PP	*p* Value		
WB BMC (g)	0.506	<0.001	−0.133	**0.284**	0.388	0.006	0.101	**0.056**	0.033	**0.569**	0.528	248.5
WB aBMD (g/cm^2^)	0.302	<0.001	0.359	0.022	−0.126	**0.476**	0.207	0.002	0.083	**0.251**	0.251	0.0804
TBLH BMC (g)	0.557	<0.001	−0.272	0.013	0.523	<0.001	0.048	**0.299**	0.025	**0.614**	0.637	200.2
TBLH aBMD (g/cm^2^)	0.395	<0.001	0.221	**0.137**	0.029	**0.862**	0.144	0.023	0.078	**0.256**	0.320	0.0735
Appendicular BMC (g)	0.584	<0.001	0.063	**0.589**	0.208	**0.114**	0.060	**0.224**	−0.004	**0.939**	0.585	162.5
Appendicular aBMD (g/cm^2^)	0.394	<0.001	0.613	<0.001	−0317	0.061	0.083	**0.188**	0.023	**0.737**	0.317	0.1032
Arms BMC (g)	0.623	<0.001	0.050	0.649	0.177	**0.156**	0.059	**0.205**	−0.084	**0.104**	0.624	35.4
Arms aBMD (g/cm^2^)	0.443	<0.001	0.402	0.006	−0.113	**0.491**	0.042	**0.493**	−0.054	**0.424**	0.353	0.0477
Legs BMC (g)	0.542	<0.001	0.063	**0.618**	0.205	**0.150**	0.057	**0.288**	0.018	**0.753**	0.513	143.5
Legs aBMD (g/cm^2^)	0.380	<0.001	0.675	<0.001	−0.394	0.022	0.098	**0.126**	0.026	**0.709**	0.301	0.1464
LS BMC (g)	0.322	<0.001	−0.121	0.453	0.279	**0.127**	0.029	**0.671**	−0.016	**0.831**	0.201	13.0
LS aBMD (g/cm^2^)	0.281	0.002	0.307	0.059	−0.076	**0.679**	0.142	0.040	0.050	**0.506**	0.196	0.1650
Pelvis BMC (g)	0.480	<0.001	−0.162	**0.222**	0.433	0.004	−0.027	**0.627**	0.080	**0.194**	0.463	60.2
Pelvis aBMD (g/cm^2^)	0.364	<0.001	0.111	**0.471**	0.062	**0.722**	0.182	0.006	0.113	**0.118**	0.264	0.1467
Trunk BMC (g)	0.256	0.002	−0.720	<0.001	0.824	<0.001	0.005	**0.933**	0.064	**0.358**	0.299	123.4
Trunk aBMD (g/cm^2^)	0.102	**0.287**	−0.340	**0.053**	0.443	0.026	0.042	**0.575**	0.099	**0.225**	0.056	0.1103
WB BMAD (g/cm^3^)	−0.267	0.003	0.156	**0.343**	0.038	**0.839**	0.296	<0.001	0.136	**0.076**	0.161	0.00546
TBLH BMAD (g/cm^3^)	−0.216	0.019	0.179	**0.289**	0.018	**0.926**	0.244	0.001	0.132	**0.094**	0.121	0.00592

WB, whole body; TBLH, total body less head; LS, lumbar spine; BMC, bone mineral content; aBMD, areal bone mineral density; BMAD, bone mineral apparent density.

**Table 7:** Linear regression of Appendicular fat-free mass index (App FFMI, kg/m^2^) together with fractional age (FA), maturity offset (MO), ethnicity (Eth), and playing position (PP) on bone mineral variables in the whole sample of elite youth soccer players (n = 193). The standardized regression coefficient (β) and its *p* value, as well as the overall adjusted coefficient of determination (AdjR^2^) and standard error of the estimate (SEE), are reported. Non-statistically significant *p* values are in bold.

Variable	β Coefficient										AdjR^2^	SEE
	App FFMI	*p* Value	FA	*p* Value	MO	*p* Value	Eth	*p* Value	PP	*p* Value		
WB BMC (g)	0.414	<0.001	−0.704	<0.001	1.049	<0.001	0.103	0.050	0.032	**0.575**	0.529	248.1
WB aBMD (g/cm^2^)	0.248	<0.001	0.017	**0.908**	0.269	**0.069**	0.208	0.002	0.083	**0.253**	0.252	0.0804
TBLH BMC (g)	0.455	<0.001	−0.901	<0.001	1.251	<0.001	0.051	**0.271**	0.025	**0.493**	0.637	200.2
TBLH aBMD (g/cm^2^)	0.324	<0.001	−0.226	**0.114**	0.546	<0.001	0.146	0.021	0.078	**0.258**	0.321	0.0735
Appendicular BMC (g)	0.480	<0.001	−0.598	<0.001	0.972	<0.001	0.062	**0.204**	−0.005	**0.929**	0.587	162.1
Appendicular aBMD (g/cm^2^)	0.326	<0.001	0.166	**0.245**	0.199	**0.157**	0.084	**0.180**	0.023	**0.741**	0.320	0.1030
Arms BMC (g)	0.509	<0.001	−0.653	<0.001	0.991	<0.001	0.062	**0.183**	−0.084	**0.100**	0.626	35.3
Arms aBMD (g/cm^2^)	0.363	<0.001	−0.099	**0.475**	0.466	0.001	0.044	**0.473**	−0.054	**0.420**	0.354	0.0477
Legs BMC (g)	0.445	<0.001	−0.551	<0.001	0.914	<0.001	0.059	**0.268**	0.018	**0.760**	0.515	143.2
Legs aBMD (g/cm^2^)	0.315	<0.001	0.243	**0.093**	0.102	**0.467**	0.099	**0.121**	0.026	**0.712**	0.304	0.1461
LS BMC (g)	0.257	<0.001	−0.481	0.002	0.698	<0.001	0.032	**0.644**	−0.016	**0.826**	0.199	13.0
LS aBMD (g/cm^2^)	0.225	0.002	−0.008	**0.960**	0.290	**0.059**	0.144	0.037	0.050	**0.509**	0.192	0.1654
Pelvis BMC (g)	0.389	<0.001	−0.702	<0.001	1.059	<0.001	−0.024	0.664	0.079	**0.198**	0.461	60.3
Pelvis aBMD (g/cm^2^)	0.295	<0.001	−0.299	0.045	0.538	<0.001	0.184	0.005	0.112	**0.119**	0.263	0.1468
Trunk BMC (g)	0.204	0.003	−1.006	<0.001	1.158	<0.001	0.007	**0.907**	0.064	**0.361**	0.297	123.5
Trunk aBMD (g/cm^2^)	0.075	**0.334**	−0.451	0.008	0.574	0.001	0.044	**0.558**	0.098	**0.226**	0.055	0.1103
WB BMAD (g/cm^3^)	−0.219	0.003	0.458	0.004	−0.311	0.047	0.295	<0.001	0.137	**0.075**	0.162	0.00546
TBLH BMAD (g/cm^3^)	−0.176	0.020	0.423	0.010	−0.265	**0.098**	0.242	0.001	0.132	**0.093**	0.121	0.00592

WB, whole body; TBLH, total body less head; LS, lumbar spine; BMC, bone mineral content; aBMD, areal bone mineral density; BMAD, bone mineral apparent density.

**Table 8:** Linear regression of fractional age (FA), body mass, stature, ethnicity (Eth), and playing position (PP) on muscle-to-bone indices in the whole sample of elite youth soccer players (n = 193). The standardized regression coefficient (β) and its *p* value, as well as the overall adjusted coefficient of determination (AdjR^2^) and standard error of the estimate (SEE), are reported. Non-statistically significant *p* values are in bold.

Variable	β Coefficient										adjR^2^	SEE
	FA	*p* Value	Body Mass	*p* Value	Stature	*p* Value	Eth	*p* Value	PP	*p* Value		
fMBU_tot_ (g/g)	0.196	0.006	0.123	**0.208**	0.162	**0.112**	0.155	0.029	0.047	**0.544**	0.128	0.00328
fMBU_app_ (g/g)	0.320	<0.001	0.011	**0.908**	0.080	**0.441**	0.069	**0.336**	−0.003	**0.917**	0.098	0.0059
MBR_WB_ (kg/kg)	−0.188	0.009	−0.077	**0.434**	−0.162	**0.117**	−0.160	0.026	0.051	**0.520**	0.103	1.873
MBR_arms_ (kg/kg)	−0.213	0.003	−0.059	**0.555**	−0.123	**0.239**	−0.119	**0.097**	0.076	**0.339**	0.089	1.604
MBR_legs_ (kg/kg)	−0.313	<0.001	0.035	**0.658**	−0.097	**0.347**	−0.063	**0.376**	−0.035	**0.658**	0.094	1.877
MBR_trunk_ (kg/kg)	0.194	0.008	−0.074	**0.462**	−0.179	**0.090**	−0.104	**0.153**	−0.080	**0.325**	0.065	5.377

fMBU, functional muscle-bone unit; MBR, muscle-to-bone ratio.

## Data Availability

The raw data supporting the conclusions of this article will be made available by the authors on request.

## Abbreviations

The following abbreviations are used in this manuscript:

FA	Fractional age
BMC	Bone mineral content
BMD	Bone mineral density
MO	Maturity offset
PP	Playing position
GK	Goalkeeper
D	Defender
M	Midfielder
F	Forward
DXA	Dual-energy X-ray absorptiometry
FFSTM	Fat-free soft tissue mass
FFMI	Fat-free mass index
fMBU	Functional muscle-bone unit
MBR	Muscle-to-bone ratio
Eth	Ethnicity
BMI	Body mass index
aBMD	Areal bone mineral density
WB	Whole body
TBLH	Total body less head region
Arms	upper limbs together
Legs	lower limbs together
Appendicular	Four limbs together
BMAD	Bone mineral apparent density
AdjR^2^	Coefficient of determination
SEE	Standard error of the estimate
PC	Partial correlation
